# Deep Reinforcement Learning Based Resource Allocation for D2D Communications Underlay Cellular Networks

**DOI:** 10.3390/s22239459

**Published:** 2022-12-03

**Authors:** Seoyoung Yu, Jeong Woo Lee

**Affiliations:** School of Electrical and Electronics Engineering, Chung-Ang University, Seoul 06974, Republic of Korea

**Keywords:** device-to-device, resource allocation, deep reinforcement learning, cellular network

## Abstract

In this paper, a resource allocation (RA) scheme based on deep reinforcement learning (DRL) is designed for device-to-device (D2D) communications underlay cellular networks. The goal of RA is to determine the transmission power and spectrum channel of D2D links to maximize the sum of the average effective throughput of all cellular and D2D links in a cell accumulated over multiple time steps, where a cellular channel can be allocated to multiple D2D links. Allowing a cellular channel to be shared by multiple D2D links and considering performance over multiple time steps require a high level of system overhead and computational complexity so that optimal RA is practically infeasible in this scenario, especially when a large number of D2D links are involved. To mitigate the complexity, we propose a sub-optimal RA scheme based on a multi-agent DRL, which operates with shared information in participating devices, such as locations and allocated resources. Each agent corresponds to each D2D link and multiple agents perform learning in a staggered and cyclic manner. The proposed DRL-based RA scheme allocates resources to D2D devices promptly according to dynamically varying network set-ups, including device locations. The proposed sub-optimal RA scheme outperforms other schemes, where the performance gain becomes significant when the densities of devices in a cell are high.

## 1. Introduction

As the demand for mobile and wireless communications grows, a large number of spectrum resources are needed to accommodate the increasing number of mobile and wireless users. Since the amount of available spectrum is limited, there are severe shortages of spectrum resources in modern wireless communication systems. Consequently, it is critical for cellular networks to support a high number of mobile users with limited spectrum resources while maintaining a high quality of services (QoS). Device-to-device (D2D) communication technology was introduced as a promising solution to resolve the spectrum shortage problem [1]. By using the D2D technology, mobile users in the proximity are able to communicate with each other directly without imposing heavy loads on cellular networks. The scope of D2D communications has been extended to vehicle-to-vehicle (V2V) and vehicle-to-everything (V2X) systems and the D2D technology is considered a key technology in fifth-generation (5G) wireless communications. Many researchers have focused on D2D technology and have conducted a large number of research activities regarding D2D communications.

Since D2D communications utilize spectrum channels, which are already occupied by cellular users, it is essential to allocate communication resources to D2D devices in a way that the performances of D2D links are improved without destroying the QoS of cellular links. Thus, the resource allocation (RA) for D2D devices in the existence of cellular devices is an inherent issue of D2D communications in theoretical and practical aspects. A large number of research studies have been conducted regarding RA for D2D communications, especially underlay cellular networks [2,3,4,5,6,7,8,9,10,11,12,13]. When different D2D links occupy distinct cellular channels, efficient one-to-one mapping techniques may be applied to RA [2]. On the other hand, in case multiple D2D links are allowed to share a cellular channel, the optimal RA problem becomes NP-hard and cannot be solved analytically, especially when a high number of wireless devices are distributed densely in a cell. Hence, sub-optimal approaches with reduced complexities have been investigated to implement RA in practical D2D communication systems [14,15,16]. In [14], a two-step resource allocation scheme was introduced to maximize the sum capacity of D2D communications. In [15], a centralized resource allocation method based on the difference of the convex function (DC) programming was proposed to solve a weighted sum rate maximization problem. In [16], an alternating channel assignment–power allocation scheme was proposed to maximize the sum rate of the cellular and D2D links.

As the scope of D2D is extended to D2D with mobility, we need a dynamic RA scheme suitable for networks with devices changing their locations continuously. It is clear that a dynamic RA requires a much higher system overhead and computational complexity than a static one because the resource allocation needs to be updated whenever a network setup, including device locations, changes. Data transmissions need to be conducted over multiple time steps for several reasons, e.g., due to the segmentation of long data frames into multiple short ones due to limited bandwidth channels. The number of time steps may be determined by data size, channel bandwidth, battery life of UEs, etc. The RA becomes more complex and hard to implement if a sequence of resources needs to be allocated over multiple time steps. Thus, a sub-optimal RA scheme with low complexity is more demanding in modern communication networks.

Recently, deep learning (DL) and reinforcement learning (RL) have received attention from a wide range of fields. DL has been actively adopted in optimization, system identification, recognition, and classification in many applications, including wireless communications. RL is a mechanism of agents that learns what to do, or how to map situations to actions, in order to maximize a reward through a trial-and-error search. Deep RL (DRL) incorporates DL into RL, in which agents make decisions from unstructured input data, where the deep Q-network (DQN) is a well-known example of DRL [17]. DRL has also been widely applied to various forms of optimization and policy determination problems, including wireless communication systems. DRL is an efficient mechanism for sequential decision-making, so it is a natural approach to apply DRL to RA over multiple time steps. Using DRL is considered a good approach to determining resources for D2D devices in a sub-optimal manner, with lower complexity in practical communication networks. In the training phase, artificial neural networks (ANNs) in agents are intensively trained for as many situations as possible. Then, in an actual operational phase, agents just observe situations and draw sub-optimal solutions to an RA problem using trained ANNs.

Many research works have applied learning techniques to RA for D2D communications [18,19,20,21,22,23,24,25,26,27,28,29,30,31,32,33,34]. As a learning principle for training RA units, DL [18,19,20,21,22], RL [23,24,25], and DRL [26,27,28,29,30,31,32,33,34] have been widely utilized. Depending on who determines the resource allocations for D2D devices, two types of RA schemes have been proposed: a centralized RA [18,19,20,23,26,27,28,31] and a decentralized RA [20,21,22,23,25,29,30,32,33,34]. In the case of DRL-based RA schemes, a single-agent framework is used for centralized RA schemes [26,27,28,31] while a multi-agent framework is used for decentralized RA schemes [21,22,23,24,25,29,30,32,33,34]. In essence, centralized single-agent RA schemes have attained a high QoS in the communication network by utilizing highly computational complexities. On the other hand, decentralized multi-agent RA schemes require low amounts of computation resulting in a degraded QoS. To obtain a high QoS with low computational complexity, we adopted a multi-agent structure to be used in the centralized RA framework, which is the main distinction from preceding works.

Various forms of DL-based RA schemes have been proposed. A hybrid power allocation scheme was proposed to maximize the sum rate of D2D users by mitigating the QoS constraint violation [18], and a channel and power allocation scheme for overlay D2D networks was proposed to maximize the sum rate of D2D pairs with a minimum rate constraint [19]. The DL framework (for the optimal RA in multi-channel cellular systems with D2D communications) was proposed to maximize the overall spectral efficiency [20], and random graph-based sparse–long short-term memory (LSTM) network for joint resource management was proposed to maximize the determinacy of latency in cellular machine-to-machine communications [21]. In [22], the RA scheme in unmanned aerial vehicle (UAV)-assisted cellular V2X (C-V2X) communications was proposed to maximize the bandwidth efficiency while satisfying the rate and latency of users.

RA schemes using RL in training phases were also proposed. An energy optimization technique was proposed in 5G wireless vehicular social networks [23], and a joint power allocation and relay selection scheme based on Q-learning was proposed to improve energy efficiency in relay-aided D2D communications underlay cellular networks [24]. A content-caching strategy based on multi-agent RL with reduced action space was introduced to maximize the expected total caching reward in mobile D2D networks [25].

An increasing number of research studies are investigating and devising RA schemes based on the DRL principle. A centralized double-DQN-based RA scheme was proposed for dynamic spectrum access in D2D communications underlay cellular networks [26], and a centralized hierarchical DRL-based method was proposed to find an optimal relay selection and power allocation strategy for 5G mmWave D2D links [27]. In [28], a DRL-based algorithm was proposed to determine the transmit power of D2D and cellular links for maximizing an overall sum-rate. In [29], each V2V link selects resources with the aid of DQN to satisfy a latency constraint and minimize the mutual interference between the infrastructure and vehicles in unicast and broadcast scenarios. A deep deterministic policy gradient (DDPG) algorithm was used for the energy efficient power control in D2D-based V2V communications [30], and an adaptive RL framework was used to select the appropriate channel selection for a non-orthogonal multiple access-unmanned aerial vehicle (NOMA-UAV) network [31]. In [32], a distributed frequency RA framework based on the multi-agent actor–critic (MAAC) was proposed. In [33], a multi-agent DRL-based distributed power control and RA algorithm was introduced to maximize the throughput of D2D and cellular users. In [34], a DRL-based joint mode selection and channel allocation algorithm was proposed in D2D communication-enabled heterogeneous cellular networks to maximize the system sum-rate in mmWave and cellular bands.

In centralized RA, a central coordinator collects information from all devices in a cell and determines the resources for all participating devices. On the other hand, in a decentralized RA, participating devices determine their own resources by using their locally obtained information. The centralized RA scheme results in a better performance than the decentralized scheme at the cost of high system overhead and high computational complexity concentrated on a central unit. On the other hand, the decentralized RA scheme results in a lower system overhead and distributed computation burden at the cost of the degradation of the communication performance. As the number of devices participating in D2D networks grows, the system overhead required to collect data from devices and deliver RA results to individual devices also increases. This results in increasing interest in decentralized RA schemes, and recently, various forms of decentralized RA schemes adopting DRL have been proposed. The basic requirement for implementing a decentralized DRL-based RA scheme involves sufficient computing capabilities from participating D2D devices because each one needs to operate its own learning mechanism, such as ANN. Vehicles and roadside infrastructures are able to supply sufficient computing power and enough space to mount high-performance devices so that V2X networks can utilize a decentralized–DRL-based RA scheme. On the other hand, personal hand-held devices do not have enough power supply and computational capabilities to run their own learning units so a decentralized DRL is not considered a viable solution for RA. Consequently, central a RA scheme still has high demands, and an advanced approach to reducing the computational complexity of DRL adopted for RA by a central coordinator is highly demanding.

In this paper, we propose a practically efficient centralized RA scheme based on a multi-agent DRL for D2D communications underlay cellular networks. We aim to present a good performance of the centralized RA scheme while reducing the computational complexity by using a multi-agent structure. Transmit power and the spectrum channel of D2D links are considered resources of D2D communications, and the objective of RA is to maximize the sum of the average effective throughput of all cellular and D2D links in a cell accumulated over multiple time steps. We obtained outage probabilities of cellular and D2D links in terms of the spectrum channel and the transmit power of the devices. Then, we define an effective throughput and formulate the optimization problem required for RA. We introduce a multi-agent DRL framework in which agents reside in a central coordinator of the cell and conduct constituent learning processes in a staggered and cyclic manner. Thanks to the segmentation of ANN into smaller ones, the proposed multi-agent DRL requires lower computational complexities in both the training phase and testing phase than the joint DRL for RA. The proposed RA scheme promptly allocates resources depending on the locations of participating devices, which vary dynamically. It was observed from simulations that the proposed DRL-based RA scheme performs well in various aspects of D2D communications underlay cellular networks. The usefulness of the proposed RA scheme is clearer in case the D2D devices are distributed more densely in a cell resulting in a higher level of mutual interferences among devices. Consequently, the proposed RA scheme is considered practically efficient in the next-generation communication network in which a high number of D2D devices with high mobility exist in cellular networks.

This paper is organized as follows. In Section 2, the system model of D2D communications underlay cellular networks is presented and the optimal resource allocation is formulated to maximize the sum of the average effective throughput of cellular and D2D links accumulated over multiple time steps. In Section 3, we provide a short introduction to the deep reinforcement learning algorithm. In Section 4, we propose a multi-agent DRL-based RA scheme, in which multiple agents conduct constituent learning in a staggered manner with a timing offset in a cyclic manner in a training phase. In Section 5, we analyze the performance of the proposed scheme in various aspects and compare it with other RA schemes. Finally, we conclude this paper in Section 6.

## 2. System Model

**Figure 1:**
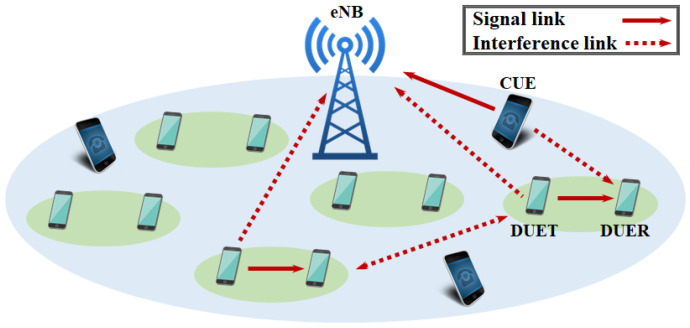
System model of D2D communications underlay cellular networks.

We consider a single cell, in which an evolved Node B (eNB), *K* cellular user equipment (CUE), and *M* D2D pairs exist, as shown in Figure 1. A D2D pair is formed by the transmitting D2D user equipment (DUET) and the receiving D2D user equipment (DUER), where D2D communications occur during a cellular uplink period. Note that RA decisions are made centrally by eNB and delivered to corresponding DUETs during a cellular downlink period. Each CUE occupies a dedicated channel while D2D links use channels already occupied by CUEs, where multiple D2D links are allowed to share a channel. We index CUE and the channel occupied by CUE as k=0,1,⋯,K−1. We also index the D2D pair and its DUET and DUER as m=0,1,⋯,M−1.

Let $s_{m}$ and ${\tilde{s}}_{k}$ denote a transmit symbol of DUET *m* and CUE *k*, respectively, each of which has the power of $p_{m}$ and ${\tilde{p}}_{k}$, respectively. We let the transmit power of each DUET be chosen out of *L* discrete values, i.e., $p_{m}\in \left\{p^{\left(1\right)},p^{\left(2\right)},\cdots ,p^{\left(L\right)}\right\}$ for each *m*. We also let $d_{xy}$ denote the distance between user equipment (UE) *x* and *y*, where $d_{xB}$ denotes the distance between UE *x* and eNB. We let ${\tilde{h}}_{kn}^{k}$ denote a small-scale fading gain of the channel between CUE *k* and DUER *n*, and let $h_{mn}^{k}$ denote a small-scale fading gain of the channel between DUET *m* and DUER *n* over the channel *k*. We suppose ${\tilde{h}}_{kn}^{k}$ and $h_{mn}^{k}$ are independent and are identically distributed (i.i.d.) zero-mean circularly symmetric complex Gaussians with unit variances. We use a log-distance model for large-scale fading in the channel between UEs *x* and *y*, which are determined by $d_{xy}^{-\alpha }$ with a path loss exponent α. We define a D2D channel access indicator $\delta_{mk}$ as $\delta_{mk}=1$ if a D2D pair *m* uses the cellular channel *k*, and $\delta_{mk}=0$ otherwise.

The received signal at DUER *m* over the channel *k* is written as

(1) $$\begin{array}{c} y_{m}^{k}=\delta_{mk}s_{m}h_{mm}^{k}\sqrt{d_{mm}^{-\alpha }}+\;\sum_{l\neq m}\delta_{lk}s_{l}h_{lm}^{k}\sqrt{d_{lm}^{-\alpha }}+{\tilde{s}}_{k}{\tilde{h}}_{km}^{k}\sqrt{d_{km}^{-\alpha }}+w_{m}, \end{array}$$

where $w_{m}$ denotes the additive noise at DUER *m*, which is a zero-mean circularly symmetric complex white Gaussian with a variance $\sigma_{w}^{2}$. The signal-to-interference-and-noise ratio (SINR) of $y_{m}^{k}$ is determined by

(2) $$\gamma_{m}^{k}=\frac{\delta_{mk}p_{m}{\left|h_{mm}^{k}\right|}^{2}d_{mm}^{-\alpha }}{\sum_{l\neq m}\delta_{lk}p_{l}|h_{lm}^{k}{|}^{2}d_{lm}^{-\alpha }+{\tilde{p}}_{k}{\left|{\tilde{h}}_{km}^{k}\right|}^{2}d_{km}^{-\alpha }+\sigma_{w}^{2}}.$$

Similarly, the SINR of the received signal at eNB over the channel *k*, denoted by $\gamma_{B}^{k}$, is defined as

(3) $$\gamma_{B}^{k}=\frac{{\tilde{p}}_{k}{\left|{\tilde{h}}_{kB}\right|}^{2}d_{kB}^{-\alpha }}{\sum_{m}\delta_{mk}p_{m}{\left|h_{mB}^{k}\right|}^{2}d_{mB}^{-\alpha }+\sigma_{w}^{2}}.$$

We declare a link outage when the achievable data rate does not meet a target rate. Let $R_{c}$ and $R_{d}$ denote target rates of the cellular link and D2D link, respectively. We also let $\gamma_{c}$ and $\gamma_{d}$ denote values of the SINR of the cellular link and D2D link, respectively, by which corresponding target rates are achieved. Note that $\gamma_{c}=2^{R_{c}}-1$ and $\gamma_{d}=2^{R_{d}}-1$, where $\log_{2}\left(1+\gamma_{c}\right)=R_{c}$ and $\log_{2}\left(1+\gamma_{d}\right)=R_{d}$. Then, $\gamma_{c}$ and $\gamma_{d}$ represent the SINR threshold for declaring outages of the cellular link and D2D link, respectively. We also let $\rho_{B}^{k}$ and $\rho_{m}^{k}$ denote the outage probabilities of the cellular link *k* and D2D link *m* over a channel *k*, respectively. Then, we obtain

(4) $$\begin{array}{cc} \rho_{B}^{k} & =\mathrm{Pr}\left\{\log\left(1+\gamma_{B}^{k}\right)<R_{c}\right\}=\mathrm{Pr}\left\{\gamma_{B}^{k}<\gamma_{c}\right\}\\ \; & =1-\exp\left(-\frac{\sigma_{w}^{2}\gamma_{c}}{{\tilde{p}}_{k}d_{kB}^{-\alpha }}\right)\cdot \;\;\;\;\prod_{m=1;\delta_{mk}=1}^{M}{\left(1+\gamma_{c}\frac{p_{m}}{{\tilde{p}}_{k}}{\left(\frac{d_{mB}}{d_{kB}}\right)}^{-\alpha }\right)}^{-1} \end{array}$$

and

(5) $$\rho_{m}^{k}=\mathrm{Pr}\left\{\gamma_{m}^{k}<\gamma_{d}\right\}=1-\exp\left(-\frac{\sigma_{w}^{2}\gamma_{d}}{p_{m}d_{mm}^{-\alpha }}\right){\left(1+\gamma_{d}\frac{{\tilde{p}}_{k}}{p_{m}}{\left(\frac{d_{km}}{d_{mm}}\right)}^{-\alpha }\right)}^{-1}\cdot \prod_{l\neq m;\delta_{lk}=1}{\left(1+\gamma_{d}\frac{p_{l}}{p_{m}}{\left(\frac{d_{lm}}{d_{mm}}\right)}^{-\alpha }\right)}^{-1},$$

whose derivations are provided in Appendix A. Note that $\rho_{m}^{k}$ is defined only when $\delta_{mk}=1$.

We define an effective throughput of the D2D link *m* over the channel *k* as a target rate multiplied by the probability of the successful transmission, i.e., $R_{d}\left(1-\rho_{m}^{k}\right)$. In the same manner, the effective throughput of the cellular link *k* is defined by $R_{c}\left(1-\rho_{B}^{k}\right)$. The goal of RA is determining the channel and transmitting power of all D2D links at each time step to maximize the cumulative sum of the average effective throughputs of the cellular and D2D links over multiple time steps *T*. Multiple D2D links are allowed to share an identical cellular channel. A D2D link occupies a single cellular channel during data transmission. Then, RA can be expressed as

(6) $$\begin{array}{cc} \; & \max_{\delta_{mk,t},p_{m,t},\forall t,k,m}\sum_{t=0}^{T-1}\{\frac{1}{K}\sum_{k=0}^{K-1}R_{c}\left(1-\rho_{B,t}^{k}\right)+\frac{1}{M}\sum_{m=0}^{M-1}\sum_{k=0}^{K-1}\delta_{mk,t}R_{d}\left(1-\rho_{m,t}^{k}\right)\}\\ \; & \;\;\mathrm{subject}\mathrm{to}\;\sum_{k=0}^{K-1}\delta_{mk,t}=1,\;\mathrm{for}\mathrm{each}\;\;m=0,\cdots ,M-1\;\;\mathrm{and}\;\;\;t=0,\cdots ,T-1\\ \; & \;\;\;\;\;0\leq \sum_{m=0}^{M-1}\delta_{mk,t}\leq M,\;\mathrm{for}\mathrm{each}\;\;k=0,\cdots ,K-1\;\mathrm{and}\;\;t=0,\cdots ,T-1, \end{array}$$

where the time step *t* is specified in $\delta_{mk}$, $p_{m}$, $\rho_{B}^{k}$, and $\rho_{m}^{k}$ as $\delta_{mk,t}$, $p_{m,t}$, $\rho_{B,t}^{k}$ and $\rho_{m,t}^{k}$, respectively, with a slight abuse of notation. Constraints in (6) imply that each D2D link utilizes only one cellular channel while a cellular channel can be used by multiple D2D links. A mathematical technique to solve (6) is not available, so we need to rely on a brute-force search approach to obtain optimal solutions of (6), which are $\delta_{mk,t}$, $p_{m,t}$ for all m,k,t. Since there exist LK possibilities of the pair of $\delta_{mk,t}$ and $p_{m,t}$ for given *m* and *t*, the overall number of possible combinations of $\delta_{mk,t}$ and $p_{m,t}$ is ${\left(LK\right)}^{MT}$. Thus, in a brute-force search, the objective function needs to be evaluated for each ${\left(LK\right)}^{MT}$ candidate to obtain an optimal solution. As a result, the optimal RA is too complex to be implemented in a practical system especially when the number of participating D2D links *M* is high. If distinct cellular channels are assigned to different D2D links, the channel allocation can be performed by a low-complexity one-to-one mapping algorithm, e.g., the Hungarian algorithm [2]. However, in case multiple D2D links are allowed to use an identical cellular channel, the high computational complexity becomes a large constraint in regard to using a brute-force-search-based RA scheme in a practical communication system. Thus, in this paper, we devise a low-complexity RA scheme based on DRL, which can be utilized in practice.

## 3. Deep Reinforcement Learning Preliminaries

Reinforcement learning (RL) is a mechanism of learning what to do, or how to map situations to actions, in order to maximize a reward through a trial-and-error search. It is known that the Markov decision process (MDP), represented by a model-free learning scheme, is useful for studying optimization problems solved by RL. MDP can formalize sequential decision-making, in which agents interact with the environment, observe states, and take actions affecting not only immediate rewards but also subsequent situations. MDP is represented by (S,A,P,R), where S is a set of states, A is a set of actions that the agent can take based on a given state, P:S×A×S→[0,1] is a transition function characterizing the probability that a given state and action are mapped to the next state, and R is a set of possible rewards obtained by an agent. If the cardinalities of S, A, and R are finite, the MDP is called a finite MDP.

In the RL, at a certain time step *t*, an agent observes a state $s_{t}\in S$ of the environment and accordingly takes an action $a_{t}\in A$ based on a policy π. The policy is a mapping from states to probabilities of selecting each possible action. Following the action $a_{t}$, the state $s_{t}$ transits to a new state $s_{t+1}$ and the agent obtains a reward $r_{t}$ and computes a return as $G_{t}=\sum_{k=0}^{\infty }\beta^{k}r_{t+k+1}$, where 0≤β≤1 is a discount factor adjusting the impact of future rewards. The agent evaluates the expected return obtained by starting from a state *s* and following a policy π, thereafter, as $v_{\pi }\left(s\right)=E\left\{G_{t}|s_{t}=s,\pi \right\}$ and the expected return obtained by starting from a state *s*, taking an action *a*, and following a policy π, thereafter, as $q_{\pi }\left(s,a\right)=E\left\{G_{t}|s_{t}=s,a_{t}=a,\pi \right\}$. Note that $v_{\pi }\left(s\right)$ and $q_{\pi }\left(s,a\right)$ are called a state-value function and an action-value function, respectively, under a policy π. Then, the agent determines the optimal policy for a given state *s* by $\pi^{*}={\mathrm{argmax}}_{\pi }v_{\pi }\left(s\right)$, through which optimal state-value function and action-value function are also defined by $v^{*}\left(s\right)=\max_{\pi }v_{\pi }\left(s\right)$ and $q^{*}\left(s,a\right)=\max_{\pi }q_{\pi }\left(s,a\right)$, respectively.

Q-learning was developed as an off-policy RL algorithm for a temporal-difference control of a finite MDP. It handles problems with stochastic transitions and resulting rewards without requiring a model of the environment. At each time step *t*, the agent staying at a state $s_{t}\in S$ selects an action $a_{t}\in A$ based on an action selection rule, which is designed to balance the behaviors of exploration and exploitation by agents. Greedy, ϵ-greedy, and soft-max methods are widely-used examples of the action selection rule. The quality of the pair of state $s_{t}$ and action $a_{t}$ is evaluated by a function Q:S×A→R, whose result Q(s,a) is called a state–action value. After taking an action, the agent observes a resultant reward $r_{t}$ and the next state $s_{t+1}$, and then updates the state–action value by

(7) $$Q\left(s_{t},a_{t}\right)\leftarrow \left(1-\mu \right)Q\left(s_{t},a_{t}\right)+\mu \left(r_{t}+\beta \max_{a}Q\left(s_{t+1},a\right)\right),$$

where μ is the learning rate. This procedure is repeated from the initial time step up to the final time step. This series of steps is called an episode. At the beginning of each episode, the environment is set to an initial state and the agent’s reward is reset to zero. We directly approximate the optimal action–value function, $q^{*}\left(s,a\right)$, by using the state–action value Q(s,a), independent of the policy being followed. It is known that in MDP, the state–action value converges with probability 1 to the optimal action–value function if each action is executed at each state during the infinite run times and the learning rate μ decays, appropriately. The optimal policy $\pi^{*}$ can be found once the optimal action–value function $q^{*}\left(s,a\right)$ is determined. After a sufficient number of updates, the state–action values for all states and actions converge.

In the case of a large state space S, evaluating the state–action values Q(s,a) for all states requires high computational complexity. We can speed up the learning process by using a function approximator, obtained from earlier experiences, to compute the state–action values. DeepMind introduced a deep Q-learning, or deep Q-network (DQN), which uses a convolutional neural network (CNN) or generally an artificial neural network (ANN) as a function approximator [17]. DQN has two phases, the (i) training phase and (ii) testing phase. In the training phase, an agent trains its state–action value approximator through a sufficient number of learning iterations. Then, the system enters a testing phase, in which the trained state–action value approximator is used to draw the best actions for a given set of observations.

**Figure 2:**
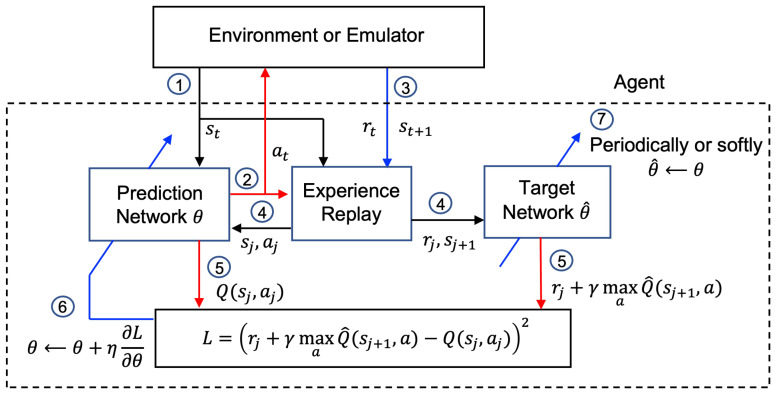
Summary of one learning iteration in the training phase of DQN, where the circled numbers denote orders of the learning process.

In the training phase of DQN, the agent utilizes two ANNs, called Q-networks, which are a prediction network and a target network. In Q-networks, states are defined by observations obtained by the agent and are fed to input nodes. With a given state *s*, the prediction network computes Q(s,a) approximately for each realization of action a∈A at each output node. The action of an agent is chosen by the action selection rule and applied to the environment or emulator. Then, a reward *r*, as well as a new state $s^{\prime }$, are obtained, and the transition vector $\left\{s,a,r,s^{\prime }\right\}$ is stored in the experience replay memory. Since observations at consecutive iterations are highly correlated, small updates of state–action values may significantly change the policy and the data distribution, which may result in the instability of RL. To overcome this problem, deep Q-learning utilizes an experience replay. Random samples of prior transition vectors are picked from an experience replay memory and used to evaluate a loss function through the prediction network and target network, where a batch of transitions may be used. This removes correlation in the observation sequence and smooths changes in the data distribution. The prediction network is updated at every time step by using the obtained loss function while the target network is updated periodically or updated softly at every time step. This process is composed of one learning iteration, which is summarized in Figure 2. After a training phase is completed by a sufficient number of iterations, the testing phase begins, in which the agent takes an action *a* corresponding to the output node of ANN having the greatest Q(s,a) for given states *s* fed to input nodes of ANN.

## 4. DRL-Based Resource Allocation for D2D Communications

We propose a DQN-based RA scheme for D2D communications underlay cellular networks. First, we consider a joint RA scheme by which resources for all D2D links are determined simultaneously. A central coordinator at eNB is considered a single agent of DQN, which conducts RA for all DUEs. We define an episode as a time duration *T* for which a sequence of data transmissions from DUET to DUER is complete. We count the time steps inside each episode, i.e., the time step *t* is defined between 0 and T−1. At time step *t*, the state $s_{t}$ is defined by locations of all UEs in the cell, indices of channel resources, and transmit power levels of D2D links at the time step *t*. Let $z_{t}$ denote the vector of locations of all UEs, i.e., CUEs, DUETs, and DUERs, and let $c_{t}$ and $p_{t}$ denote vectors of allocated channel indices and transmit power levels of all D2D links at time step *t*, respectively. Then, the state is expressed as

(8) $$s_{t}=\left\{z_{t},c_{t},p_{t}\right\}.$$

We define the action ($a_{t}$) at time step *t* by the determination of the transmit power levels and channel indices to all D2D links at time step *t*. The instantaneous reward at time step *t*, denoted by $r_{t}$, is defined as the sum of the average effective throughput of D2D and cellular links in the cell at time step *t*, i.e.,

(9) $$r_{t}=\frac{1}{K}\sum_{k=0}^{K-1}R_{c}\left(1-\rho_{B,t}^{k}\right)+\frac{1}{M}\sum_{m=0}^{M-1}\sum_{k=0}^{K-1}\delta_{mk,t}R_{d}\left(1-\rho_{m,t}^{k}\right),$$

and the accumulated reward up to the time step t−1, denoted by ${\tilde{r}}_{t}$, is obtained as

(10) $${\tilde{r}}_{t}=\sum_{i=0}^{t-1}r_{i},\;t\leq T.$$

The reward accumulated over the time steps in an episode will be called a *benefit* and expressed as

(11) $${\tilde{r}}_{T}=\sum_{t=0}^{T-1}r_{t}.$$

The goal of RA is to maximize the benefits under existing constraints as introduced in (6).

**Figure 3:**
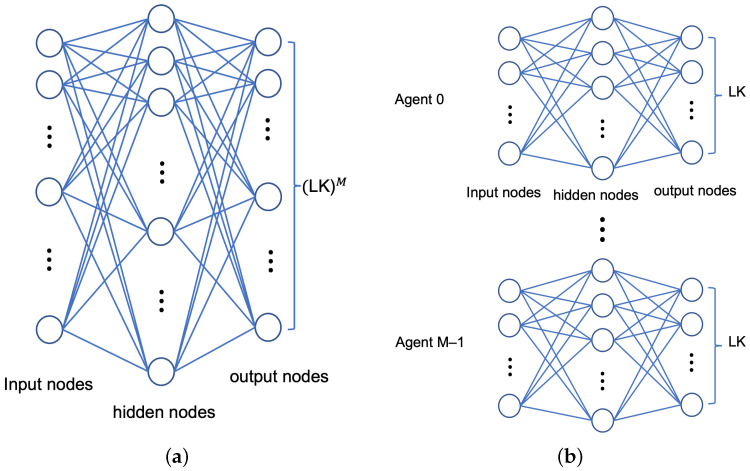
Structure of artificial neural network (ANN) implemented in DQN. (**a**) Joint DQN, (**b**) Multi-agent DQN.

In DQN, the state $s_{t}$ is fed to input nodes of ANN and each output node of ANN is dedicated to each action. As introduced in (8), a state is defined by a (K+4M)-tuple vector, which are (K+2M) entries of $z_{t}$ and *M* entries for each of $c_{t}$ and $p_{t}$. Each *M* D2D link can be allocated a channel index and a power level out of *K* and *L* possible values, respectively, so that there exist ${\left(LK\right)}^{M}$ possible realizations of action at each time step. It follows that ANNs in the prediction network and target network have K+4M input nodes and ${\left(LK\right)}^{M}$ output nodes, as shown in Figure 3a. Suppose ANN has $L_{h}$ hidden layers, each of which has $N_{h}$ nodes. We also suppose neighboring layers are fully connected. In the testing phase, ANN performs the forward propagation from the input layer to the output layer. We consider the computational complexity of the forward propagation of ANN in terms of floating point operation (FLOP) [35]. A forward propagation from a layer with $N_{i}$ nodes to a layer with $N_{j}$ nodes requires approximately $2N_{i}N_{j}$ FLOPs where the computational complexities of the activation functions of nodes are negligible. Then, FLOPs required for forward propagation over ANN is approximately $2\left\{\left(K+4M\right)N_{h}+L_{h}N_{h}^{2}+N_{h}{\left(LK\right)}^{M}\right\}$. In the training phase, ANN is updated through multiple pairs of forward and backward propagations. It is known that FLOPs of backward propagation are typically 2–3 times the FLOPs of forward propagation [35]. Thus, it is sufficient to focus on forward propagation when comparing computational complexities of ANNs. When *L*, *K*, and *M* are high, ${\left(LK\right)}^{M}$ is much higher than K+4M and $N_{h}$ so that the computational complexity of ANN is dominated by $N_{h}{\left(LK\right)}^{M}$. Since $N_{h}{\left(LK\right)}^{M}$ FLOPs are too high to be executed in real time, the RA scheme based on a single-agent DQN is practically infeasible with high *L*, *K*, and *M*.

To resolve this problem, we utilize a structure of multi-agent DQN, in which each agent corresponds to each D2D link and operates its own DQN. Note that agents exist physically in a central coordinator at eNB. Agents have ANNs in prediction and target networks with segmented structures as depicted in Figure 3b. Agents share a state, which is identical to the state of a single-agent DQN defined in (8). The action of an agent is reduced to the allocation of transmit power and spectrum channel of the corresponding D2D link only. Thus, the action chosen by the agent *m* at time step *t*, denoted by $a_{t}^{m}$, is defined by

(12) $$a_{t}^{m}=\left\{c_{t}^{m},p_{t}^{m}\right\}$$

where $c_{t}^{m}\in \left\{0,\cdots ,K-1\right\}$ and $p_{t}^{m}\in \left\{0,\cdots ,L-1\right\}$ denote the index of channel and transmit power level of the D2D link *m* at the time step *t*, respectively. Since each D2D link has LK possible realizations of action, the ANN in each DQN has LK output nodes as shown in Figure 3b, where the number of input nodes remains as K+4M. Then, the overall FLOPs required for forward propagations over *M* segmented ANNs is approximately $2M\left\{\left(K+4M\right)N_{h}+L_{h}N_{h}^{2}+N_{h}\left(LK\right)\right\}$. If $LKM>>N_{h}$, the computational complexity of multiple-segmented ANNs is dominated by $N_{h}LKM$, which is $M/{\left(LK\right)}^{M-1}$ of the complexity of a single-agent ANN. On the other hand, if $N_{h}>>LKM$, the complexity is dominated by $ML_{h}N_{h}^{2}$, which is $ML_{h}N_{h}/{\left(LK\right)}^{M}$ of the complexity of a single-agent ANN. In both cases, a significant level of complexity reduction is observed by using the multi-agent DQN.

The learning process of multi-agent RL is executed as described below. Let us define constituent learning as a sequence of operations by a single agent, i.e., observing a state *s*, taking an action *a*, observing a reward *r* and a new state $s^{\prime }$, updating weights of prediction/target networks θ and $\theta^{\prime }$. If all agents conduct constituent learning simultaneously, it is impossible to evaluate explicitly the influence of individual agent actions on the change of the environment. Since the reward and the next state fed back to each agent do not reflect explicitly the contribution of the corresponding agent to the environment, the multi-agent RL may not converge well or may not improve performance through iterations. Thus, it is a reasonable approach to devise a sequential operation of constituent learning by multiple agents.

**Figure 4:**
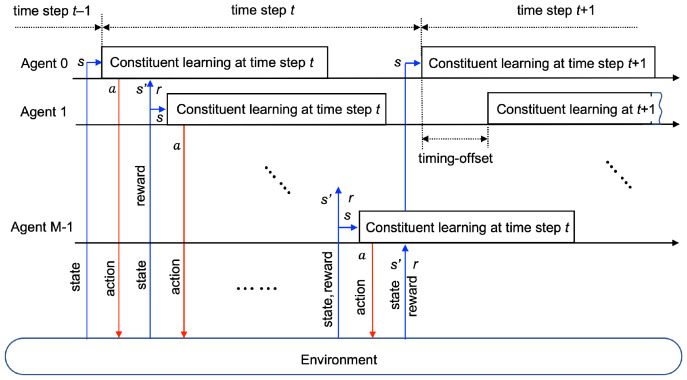
Learning of multiple agents in a cyclic manner.

We let agents conduct constituent learning in a cyclic manner with the timing offset as depicted in Figure 4. Without loss of generality, labeling agents is based on the order of performing constituent learning. This order is randomly selected at the beginning of the training phase and is maintained. After completing one constituent learning procedure, each agent keeps idling until its turn comes around again, by which each agent performs constituent learning periodically. We define a time step as a time interval corresponding to a period of learning by agent 0 as depicted in Figure 4. All UEs may change their locations at the beginning of every time step. After an agent *m* takes an action, a new state is observed by this agent as a result of environmental change. This newly observed state is also used as a state initiating constituent learning by the next agent, i.e., $s_{t}^{m}={s^{\prime }}_{t}^{m-1}$, if m≠0. On the other hand, agent 0 does not use a newly observed state ${s^{\prime }}_{t-1}^{M-1}$ of agent M−1 as an initial state $s_{t}^{0}$ because UEs may change locations at the beginning of the time step. All agents complete starting constituent learning processes within a time step. Note that $s_{t}^{m}\neq {s^{\prime }}_{t-1}^{m}$ due to the existence of the idling period between adjacent constituent learning of each agent, which is a modification from the conventional DQN. In this manner, the overall cyclic learning procedure is operated during a training phase. The collection of constituent learning of all agents composes a learning iteration. We let $s_{t}^{m}$, $a_{t}^{m}$, $r_{t}^{m}$, and ${s^{\prime }}_{t}^{m}$ denote state, action, reward, next state of the agent *m* at time step *t*. Even at the same time step, different agents have different values for these variables.

The learning procedure of each agent over multiple time steps and episodes is described as follows, which is also summarized in Algorithm 1. For a simple description, we focus on the operation of a specific agent indexed by *m*, where m=0,⋯,M−1. This corresponds to a single timeline of a single agent in Figure 4. First, we initialize weights of prediction networks $\theta_{m}$ by randomly generated small numbers, and set weights of target networks as $\theta_{m}^{{}^{\prime }}=\theta_{m}$. Experience replay memory $D_{m}$ is initialized by running a random policy. The agent *m* observes a state $s_{t}^{m}$ and takes an action $a_{t}^{m}$ based on the ϵ-greedy policy as an action selection rule to affect the environment. This implies that the agent *m* selects an action $a_{t}^{m}$ resulting in the maximum state–action value $Q(s_{t}^{m},a_{t}^{m};\theta_{m})$ with probability (1−ϵ) or selects an action randomly from other candidates with probability ϵ. Note that we use the expression Q(s,a;θ) to declare that the state–action value Q(s,a) is obtained by ANN with weights θ. Affected by action $a_{t}^{m}$, the environment changes and the agent *m* obtains a reward $r_{t}^{m}$ by (9) and observes a next state ${s^{\prime }}_{t}^{m}$. The transition vector $\left\{s_{t}^{m},a_{t}^{m},r_{t}^{m},{s^{\prime }}_{t}^{m}\right\}$ is stored in the experience replay memory $D_{m}$. The batch of transition vectors, which have been previously stored in $D_{m}$, are sampled randomly and used to evaluate a loss function as the following. Suppose $\left\{s_{j}^{m},a_{j}^{m},r_{j}^{m},{s^{\prime }}_{j}^{m}\right\}$ is one sample included in a batch B picked up from $D_{m}$, where we use subscript *j* to represent an index at which the transition vector is stored in $D_{m}$ with a slight abuse of notation. The predicted state–action value $Q(s_{j}^{m},a_{j}^{m};\theta_{m})$ is obtained from the output node corresponding to the action $a_{j}^{m}$ in ANN of prediction network with weight $\theta_{m}$. The target state–action value $y_{j}^{m}$ is obtained by a target network with weight $\theta_{m}^{\prime }$ as

(13) $$y_{j}^{m}=\left\{\begin{array}{cc} r_{j}^{m}, & \mathrm{if}\;s_{j}^{m}\;\mathrm{is}\;a\;\mathrm{terminal}\;\mathrm{state}\\ r_{j}^{m}+\beta \max_{a}Q({s^{\prime }}_{j}^{m},a;\theta_{m}^{{}^{\prime }}), & \mathrm{otherwise}. \end{array}\right.$$

Then, the loss function $L^{m}$ is computed as the mean squared error between the target state–action value and predicted state–action value by

(14) $$L^{m}=\frac{1}{\left|B\right|}\sum_{j\in B}{\left(y_{j}^{m}-Q\left(s_{j}^{m},a_{j}^{m};\theta_{m}\right)\right)}^{2},$$

where |B| represents the size of batch B. We update the weights of the prediction network $\theta_{m}$ by using a stochastic gradient descent algorithm as

(15) $$\theta_{m}\leftarrow \theta_{m}+\eta \frac{\partial L^{m}}{\partial \theta_{m}},$$

where η is a learning rate, and update weights of target network $\theta_{m}^{\prime }$ softly as

(16) $$\theta_{m}^{{}^{\prime }}\leftarrow \left(1-\tau \right)\theta_{m}^{{}^{\prime }}+\tau \theta_{m},$$

where τ<<1. We repeat the above process over the time steps in each episode and repeat the whole process over *E* episodes.

After the training phase is completed through multiple episodes as introduced above, the RA scheme enters a testing phase which corresponds to an actual operation of DUEs underlay cellular networks. At every time step, observation obtained by eNB is used by each agent as a state, which is input to the trained prediction network. Then, the action resulting in the maximum state–action value at the output nodes of the prediction network is chosen for each agent. The chosen action is reported to DUET and used as resources for the corresponding D2D communication. In the testing phase, resource allocation for all agents may be executed simultaneously, not in a staggered manner, at each time step. The environment is influenced by D2D communications performed in this manner, and new observation is obtained by eNB. This procedure repeats over all time steps of the testing phase.
**Algorithm 1** Training Phase of Agent *m* in a Multi-Agent DRL-Based RA**Initialization:**Randomly initialize weights of prediction network $\theta_{m}$.Initialize weights of the target network by $\theta_{m}^{{}^{\prime }}\leftarrow \theta_{m}$.Initialize experience replay memory $D_{m}$.**for** e=1,⋯,E **do**     **for** t=0,⋯,T−1  **do**        Observe state $s_{t}^{m}=\left\{z_{t}^{m},c_{t}^{m},p_{t}^{m}\right\}$.        Determine action $a_{t}^{m}=\left(c_{t}^{m},p_{t}^{m}\right)$ based on the action selection rule.        Report the chosen action to DUET and DUET takes an action accordingly.        Observe reward $r_{t}^{m}$ and next state ${s^{\prime }}_{t}^{m}$.        Store the transition vector $\left\{s_{t}^{m},a_{t}^{m},r_{t}^{m},{s^{\prime }}_{t}^{m}\right\}$ in $D_{m}$.        Randomly sample the batch of transition vectors B from $D_{m}$.        Obtain $Q(s_{j}^{m},a_{j}^{m};\theta_{m})$ from the prediction network.        Obtain $y_{j}^{m}$ by (13) in the target network.        Compute the loss function $L^{m}$ by (14).        Update $\theta_{m}$ and $\theta_{m}^{{}^{\prime }}$ by (15) and (16), respectively.     **end for****end for**

## 5. Numerical Results

We consider a single circular-shaped cell, in which eNB is located at the center, and *K* CUEs as well as *M* DUE pairs exist, where DUERs are placed around the corresponding DUETs within a distance of 5 [m]. The distribution of CUE and DUET in the cell and the distribution of DUER around the DUET follow the binomial point process (BPP) model [36]. All UEs change their locations at every time step. Simulation parameters used in numerical experiments are listed in Table 1 and hyperparameters used for DRL are listed in Table 2. Simulation software used for numerical experiments were Python 3.6.12 and PyTorch 1.4.0. The transmit power of each CUE was determined such that the corresponding SNR at eNB resulted in an outage probability of 0.1%. We consider various values for $R_{c}$ and ${\bar{r}}_{c}$ for the analysis of RA schemes in various aspects.

**Figure 5:**
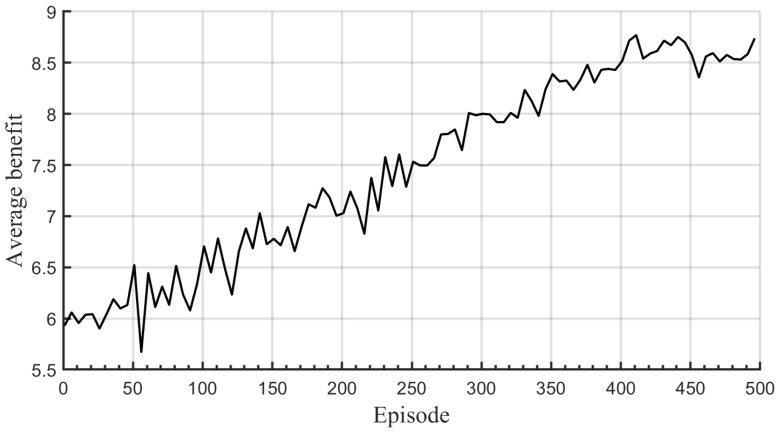
Evolution of average benefit for $R_{c}=8$ [bit/s/Hz], ${\bar{r}}_{c}=300$ [m] and M=16, where average values of benefit over every 5 episodes are plotted.

Each ANN in the prediction and target networks has five fully connected layers, the middle three of which are hidden layers. Each hidden layer has 300 neurons equipped with the ReLU activation function; a stochastic gradient descent optimizer is used for updating the weight of ANNs. Experiences replaying memories are initially filled with experiences obtained by running random policies. The training phase is completed in 5000 iterations (500 episodes and 10 iterations/episode), and the ϵ-greedy policy with linear annealing is applied as an action selection rule. It is observed from Figure 5 that DQNs are updated well during the training phase.

We evaluate the performances of RA schemes in terms of average benefits and compare performances of the proposed DRL-based RA, random RA, and greedy RA schemes. A random RA allocates the spectrum channel and transmits the power of the D2D links randomly. In a greedy RA, the channel and transmit power of D2D links are determined through a greedy search to maximize the sum of the average effective throughput for each time step. We consider a scenario that every time step, locations of UEs change, and resources of all D2D pairs are allocated simultaneously.

**Figure 6:**
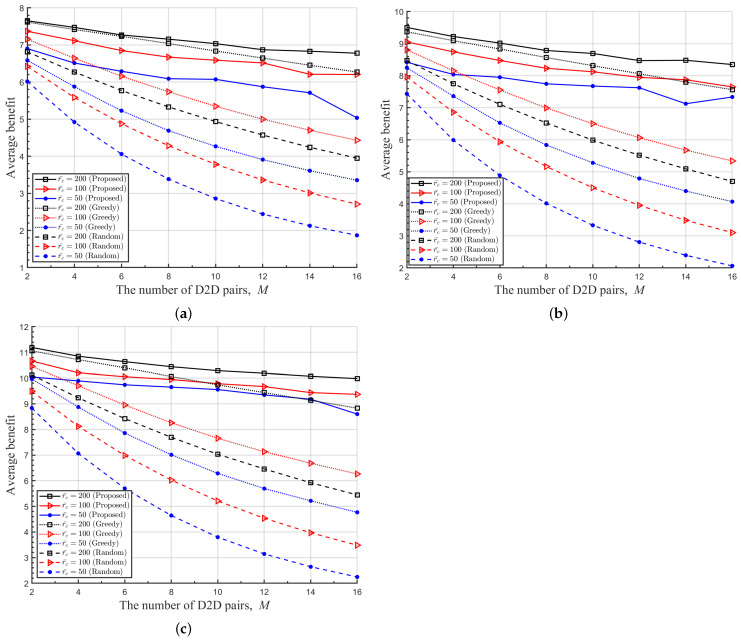
Average benefit of the proposed and other RA schemes with respect to *M* for various ${\bar{r}}_{c}$. (**a**) $R_{c}=6\;\left[\mathrm{bits}/s/\mathrm{Hz}\right]$, (**b**) $R_{c}=8\;\left[\mathrm{bits}/s/\mathrm{Hz}\right]$, (**c**) $R_{c}=10\;\left[\mathrm{bits}/s/\mathrm{Hz}\right]$.

In Figure 6, we plot the average benefits obtained by various RA schemes under comparison with respect to the number of D2D pairs *M* existing in the cell, where various radii of cell ${\bar{r}}_{c}$ and target rates of cellular link $R_{c}$ are considered. It is observed from Figure 6 that the proposed DRL-based RA scheme shows better performance than others in all situations. As the number of D2D links *M* in the cell grows, all RA schemes show lower average benefits due to resulting severer mutual interference among UEs. However, the performance degradation of the proposed DRL-based RA scheme is less sensitive to the growth of *M* than other RA schemes. Thus, the performance gain of the proposed RA scheme over others becomes significant as the number of D2D pairs in the cell increases. It is also observed that the performance gain of the proposed DRL-based RA scheme over others increase as the radius of cell ${\bar{r}}_{c}$ decrease. From these observations, it is obviously inferred that the proposed RA scheme is quite useful especially when DUEs are distributed densely in a cell and suffer from a high level of mutual interference from other UEs. Since the demand for D2D communications is continuously growing, the proposed RA scheme would be a meaningful solution to resolve the spectrum shortage problem in the next-generation wireless communication systems.

**Figure 7:**
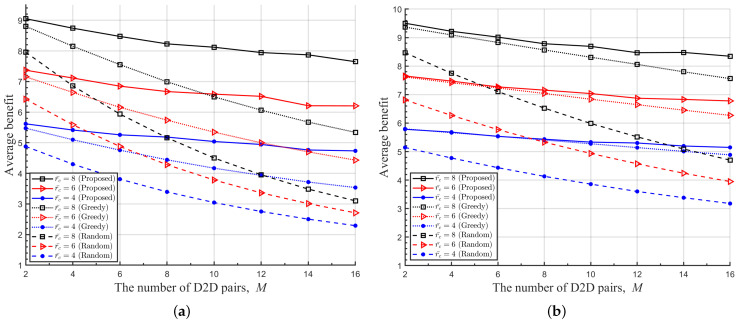
Average benefit of the proposed and other RA schemes with respect to *M* for various $R_{c}$. (**a**) ${\bar{r}}_{c}$ = 100 [m], (**b**) ${\bar{r}}_{c}$ = 200 [m].

It is additionally observed that the proposed DRL-based RA scheme attains highly improved average benefit with higher $R_{c}$ compared with other RA schemes, which is clear from Figure 7. This is explained by the property that the proposed RA scheme balances well effective throughputs of both D2D links and cellular links, while a greedy RA scheme has a priority in maintaining the QoS of D2D links at a required level. Consequently, the proposed DRL-based RA scheme obtains a significant performance gain over others in case the DUEs are distributed densely in a cell and CUE has a higher target rate than DUE.

**Figure 8:**
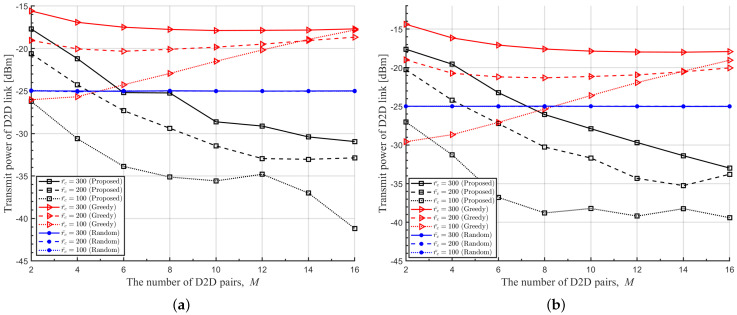
Average transmit power of DUET with respect to *M* for various ${\bar{r}}_{c}$. (**a**) $R_{c}=6\;\left[\mathrm{bits}/s/\mathrm{Hz}\right]$, (**b**) $R_{c}=8\;\left[\mathrm{bits}/s/\mathrm{Hz}\right]$.

**Figure 9:**
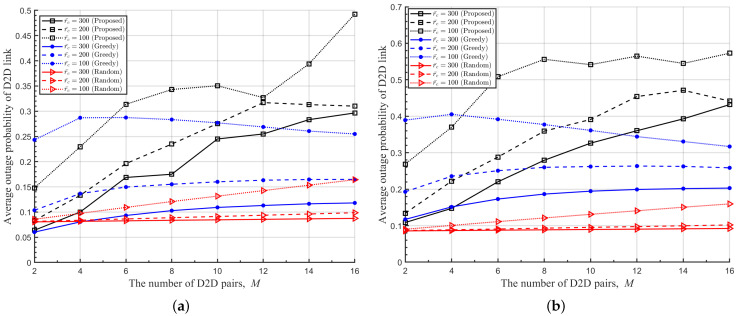
Average outage probability of D2D links with respect to *M* for various ${\bar{r}}_{c}$. (**a**) $R_{c}=6\;\left[\mathrm{bits}/s/\mathrm{Hz}\right]$, (**b**) $R_{c}=8\;\left[\mathrm{bits}/s/\mathrm{Hz}\right]$.

**Figure 10:**
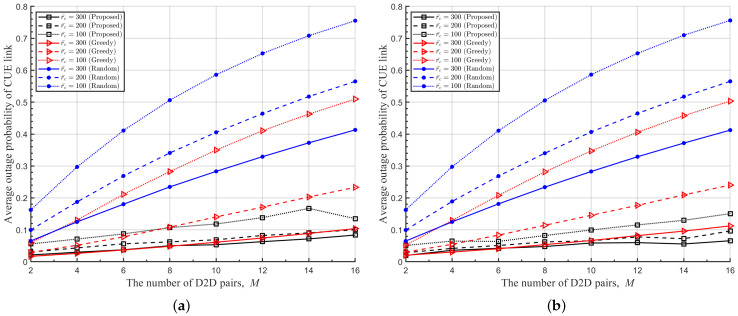
Average outage probability of cellular links with respect to *M* for various ${\bar{r}}_{c}$. (**a**) $R_{c}$ = 6 [bits/s/Hz], (**b**) $R_{c}$ = 8 [bits/s/Hz].

**Figure 11:**
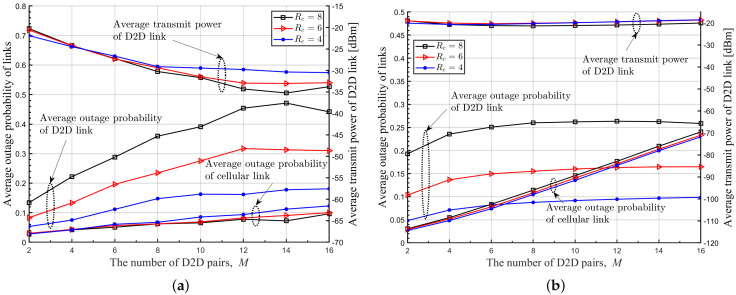
Average outage probabilities of cellular and D2D links and average transmit power of DUET with respect to *M* for various $R_{c}$, where ${\bar{r}}_{c}$ = 200 [m]. (**a**) Proposed DRL-based RA, (**b**) Greedy RA.

In Figure 8, Figure 9 and Figure 10, we plot the average transmit power of DUETs, the average outage probability of D2D links, and the average outage probability of cellular links with respect to *M*, respectively, where various $R_{c}$ and ${\bar{r}}_{c}$ are considered. It is observed that the proposed DRL-based RA scheme adapts the transmitting power sensitively with respect to *M* and ${\bar{r}}_{c}$, to achieve high benefits while maintaining the QoS of the cellular links. For larger *M* and smaller ${\bar{r}}_{c}$, the proposed RA scheme prevents benefits from decreasing by using a lower transmitting power of DUET at the cost of a higher outage probability of the D2D links. The overall performance is maintained at the sacrifice of the D2D link because the cellular link has a higher contribution to the overall performance than the D2D link when $R_{c}$ is high. Although a greedy RA scheme also adapts the transmit power of DUET depending on *M*, higher transmit power is allocated to D2D links with growing *M* and thus the average outage probability of D2D links is maintained at the cost of increasing the average outage probability of cellular links. For higher $R_{c}$, this way of power allocation results in higher degradation in the average benefit so the performance is outperformed further by the proposed RA scheme. Figure 11 shows clearly that a higher ratio of $R_{c}/R_{d}$ results in lower transmitting power of DUET and, thus, a higher outage probability of D2D links by the proposed DRL-based RA scheme, which is different from a greedy RA scheme. The performance gain of the proposed RA scheme over others comes from the fact that each agent was trained to know implicitly how other agents will act at the next time step for a given set of observations. In other RA schemes, on the other hand, D2D links determine their resources only depending on the current observation. Since each agent in the proposed RA scheme takes an action based on the prediction of other agents’ future actions, the proposed RA scheme works much better than others especially in case a high level of mutual interference among UEs exists.

The proposed DRL-based RA scheme allocates adaptively communication resources depending on the locations of UEs, the cell size, and the target rate of the cellular link by using the pre-trained allocation rule, which enables a fast RA in the actual operation of communication networks.

## 6. Conclusions

We proposed a DRL-based RA scheme for the communications of D2D pairs underlaying cellular networks, where the spectrum channel for the D2D link and the transmit power of DUET are considered communication resources to be determined. Multiple D2D pairs are allowed to share a cellular channel, which results in high computational complexity when determining channels for D2D links. Moreover, ANNs used in DRL for joint RA have a high number of output nodes resulting in high computational complexity. To resolve this problem, a multi-agent DQN is adopted in the proposed scheme, resulting in segmented ANNs and, thus, reduced computational complexity. In the testing phase corresponding to the period of actual operation, the proposed scheme allocates communication resources adaptively in a real-time manner depending on the network setup by using the pre-trained ANNs. The proposed RA scheme outperforms others, especially when UEs are distributed densely, resulting in a high level of mutual interferences and the QoS of the cellular link has a higher priority than the D2D link.

## Figures and Tables

**Table 1:** Simulation parameters for D2D communications underlay cellular networks.

Parameter	Value
Number of CUEs, *K*	4
Number of D2D pairs, *M*	2, 4, 6, 8, 10, 12, 14, 16
Radius of the cell, ${\bar{r}}_{c}$	50, 100, 200, 300 [m]
Path loss exponent, α	3.5
Number of transmit power levels, *L*	8
Minimum and maximum transmit power	−60 [dBm] and 10 [dBm]
Power gap between the adjacent power levels	10 [dBm]
Noise power, $\sigma_{w}^{2}$	−104 [dBm]
Target rate of the cellular link, $R_{c}$	4, 6, 8, 10 [bits/s/Hz]
Target rate of the D2D link, $R_{d}$	2 [bits/s/Hz]

**Table 2:** Hyperparameters for DRL.

Parameter	Value
Learning rate, η	0.001
Discount factor, β	0.1
Length of the episode, *T*	10
Number of episodes, *E*	500
Batch size, |B|	64
Experience replay memory size	50,000
Initial and Final exploration rate, ϵ	1.0 and 0.1
Soft target update parameter, τ	0.01

## Data Availability

Not applicable.

## Appendix A. Derivation of Outage Probabilities

Consider the exponentially distributed random variable $\alpha_{i}$, i=0,⋯,N, whose probability density function (pdf) is given by $f_{\alpha_{i}}\left(a_{i}\right)=\lambda_{i}e^{-\lambda_{i}a_{i}}$ with mean $\lambda_{i}^{-1}$ and variance $\lambda_{i}^{-2}$. Let us define γ as

(A1) $$\gamma =\frac{\alpha_{0}}{\sum_{i=1}^{N}\alpha_{i}+b}.$$

Then, its cumulative distribution function (CDF) is obtained by

(A2) $$\begin{array}{cc} \mathrm{Pr}\left\{\gamma <r\right\}\; & =\mathrm{Pr}\left\{\frac{\alpha_{0}}{\sum_{i=1}^{N}\alpha_{i}+b}<r\right\}\\ \; & =\mathrm{Pr}\left\{\alpha_{0}^{\prime }<\sum_{i=1}^{N}\alpha_{i}+b\right\}\\ \; & =\int_{0}^{\infty }\cdots \int_{0}^{\infty }\left(\int_{0}^{\sum_{i=1}^{N}a_{i}+b}f_{\alpha_{0}^{\prime }}\left(a_{0}^{\prime }\right)da_{0}^{\prime }\right)\prod_{i=1}^{N}f_{\alpha_{i}}\left(a_{i}\right)da_{i}. \end{array}$$

where $\alpha_{0}^{\prime }=\frac{\alpha_{0}}{r}$ and $f_{\alpha_{0}^{\prime }}\left(a_{0}^{\prime }\right)=\lambda_{0}^{\prime }e^{-\lambda_{0}^{\prime }a_{0}^{\prime }}$ with $\lambda_{0}^{\prime }=r\lambda_{0}$. Since $\int_{0}^{\sum_{i=1}^{N}a_{i}+b}f_{\alpha_{0}^{\prime }}\left(a_{0}^{\prime }\right)da_{0}^{\prime }=1-e^{-\lambda_{0}^{\prime }\left(\sum_{i=1}^{N}a_{i}+b\right)}$, we rewrite and expand (A2) as

(A3) $$\begin{array}{cc} \mathrm{Pr}\left\{\gamma <r\right\}\; & =\int_{0}^{\infty }\cdots \int_{0}^{\infty }\left(1-e^{-\lambda_{0}^{\prime }\left(\sum_{i=1}^{N}a_{i}+b\right)}\right)\prod_{i=1}^{N}f_{\alpha_{i}}\left(a_{i}\right)da_{i}\\ \; & =1-\int_{0}^{\infty }\cdots \int_{0}^{\infty }e^{-\lambda_{0}^{\prime }\left(\sum_{i=1}^{N}a_{i}+b\right)}\prod_{i=1}^{N}\lambda_{i}e^{-\lambda_{i}a_{i}}da_{i}\\ \; & =1-e^{-\lambda_{0}^{\prime }b}\int_{0}^{\infty }\cdots \int_{0}^{\infty }\prod_{i=1}^{N}\lambda_{i}e^{-\left(\lambda_{0}^{\prime }+\lambda_{i}\right)a_{i}}da_{i}\\ \; & =1-e^{-\lambda_{0}^{\prime }b}\prod_{i=1}^{N}\int_{0}^{\infty }\lambda_{i}e^{-\left(\lambda_{0}^{\prime }+\lambda_{i}\right)a_{i}}da_{i}. \end{array}$$

Since $\int_{0}^{\infty }\lambda_{i}e^{-\left(\lambda_{0}^{\prime }+\lambda_{i}\right)a_{i}}da_{i}=\frac{\lambda_{i}}{\lambda_{0}^{\prime }+\lambda_{i}}$, we can rewrite (A3) as

(A4) $$\begin{array}{cc} \mathrm{Pr}\left\{\gamma <r\right\}\; & =1-e^{-\lambda_{0}^{\prime }b}\prod_{i=1}^{N}\frac{\lambda_{i}}{\lambda_{0}^{\prime }+\lambda_{i}}=1-e^{-\lambda_{0}rb}\prod_{i=1}^{N}\frac{\lambda_{i}}{\lambda_{0}r+\lambda_{i}}\\ \; & =1-e^{-\frac{b}{\mu_{0}}r}\prod_{i=1}^{N}{\left(1+\frac{\mu_{i}}{\mu_{0}}r\right)}^{-1}, \end{array}$$

where $\mu_{i}=E\left\{\alpha_{i}\right\}=\lambda_{i}^{-1}$.
